# Preliminary study on DCE-MRI radiomics analysis for differentiation of HER2-low and HER2-zero breast cancer

**DOI:** 10.3389/fonc.2024.1385352

**Published:** 2024-08-15

**Authors:** Liang Yin, Yun Zhang, Xi Wei, Zakari Shaibu, Lingling Xiang, Ting Wu, Qing Zhang, Rong Qin, Xiuhong Shan

**Affiliations:** ^1^ Department of Breast Surgery, Jiangsu University Affiliated People's Hospital, Zhenjiang, China; ^2^ Zhenjiang Clinical Medical College of Nanjing Medical University, Zhenjiang, China; ^3^ School of Medical Imaging, Jiangsu University, Zhenjiang, China; ^4^ Department of Radiology, Jiangsu University Affiliated People's Hospital, Zhenjiang, China; ^5^ Department of Pathology, Jiangsu University Affiliated People’s Hospital, Zhenjiang, China; ^6^ School of Medicine, Jiangsu University, Zhenjiang, Jiangsu, China; ^7^ Department of Ultrasound, Jiangsu University Affiliated People’s Hospital, Zhenjiang, China; ^8^ Department of Medical Oncology, Jiangsu University Affiliated People's Hospital, Zhenjiang, China

**Keywords:** HER2-low, HER2-zero, breast cancer, DCE-MRI, radiomics analysis, nomogram

## Abstract

**Purpose:**

This study aims to evaluate the utility of radiomic features from dynamic contrast-enhanced magnetic resonance imaging (DCE-MRI) in distinguishing HER2-low from HER2-zero breast cancer.

**Patients and methods:**

We retrospectively analyzed 118 MRI cases, including 78 HER2-low and 40 HER2-zero patients confirmed by immunohistochemistry or fluorescence *in situ* hybridization. From each DCE-MRI case, 960 radiomic features were extracted. These features were screened and reduced using intraclass correlation coefficient, Mann-Whitney U test, and least absolute shrinkage to establish rad-scores. Logistic regression (LR) assessed the model’s effectiveness in distinguishing HER2-low from HER2-zero. A clinicopathological MRI characteristic model was constructed using univariate and multivariate analysis, and a nomogram was developed combining rad-scores with significant MRI characteristics. Model performance was evaluated using the receiver operating characteristic (ROC) curve, and clinical benefit was assessed with decision curve analysis.

**Results:**

The radiomics model, clinical model, and nomogram successfully distinguished between HER2-low and HER2-zero. The radiomics model showed excellent performance, with area under the curve (AUC) values of 0.875 in the training set and 0.845 in the test set, outperforming the clinical model (AUC = 0.691 and 0.672, respectively). HER2 status correlated with increased rad-score and Time Intensity Curve (TIC). The nomogram outperformed both models, with AUC, sensitivity, and specificity values of 0.892, 79.6%, and 82.8% in the training set, and 0.886, 83.3%, and 90.9% in the test set.

**Conclusions:**

The DCE-MRI-based nomogram shows promising potential in differentiating HER2-low from HER2-zero status in breast cancer patients.

## Introduction

Breast cancer (BC) is the most prevalent form of cancer globally and remains the leading cause of cancer-related deaths in women (1). This disease comprises diverse biological entities with different prognoses and oncogenic drivers (2, 3). BC can be categorized into five intrinsic subtypes using the PAM50 gene expression profile, while traditional histological markers define four fundamental clinical subtypes with prognostic value (4–8). Notably, HER2-positive tumors, representing 15% of invasive BC, have a more aggressive clinical course and poorer prognosis (9, 10). However, advancements in HER2 therapies have improved the history and prognosis of these tumors (11).

Recently, tumors with HER2 immunohistochemistry (IHC) 1+ or 2+ expression levels but negative *in situ* hybridization (ISH) have been classified as HER2-low BC. Studies, such as NSABP B-47, have shown that conventional anti-HER2 therapies do not effectively treat HER2-low BC (12). Yet, clinical data suggest that antibody-conjugate drugs like trastuzumab deruxtecan and trastuzumab duocarmazine may benefit patients with low HER2 expression, expanding beyond traditional HER2-positive tumors (13, 14). The introduction of antibody-conjugate drugs has reshaped the HER2 landscape, as seen in studies like DESTINY Breast04, where trastuzumab deruxtecan demonstrated efficacy in HER2-low BC (13). Identifying HER2-low status early in the disease course is crucial for tailoring treatment strategies, especially in therapy-resistant, hormone receptor-negative tumors (15, 16). Early detection of HER2-low status during the disease process is critical for optimizing and customizing treatment strategies. However, the restricted sampling of potentially heterogeneous lesions during biopsies can result in inconsistencies and inaccuracies in distinguishing between HER2-low and HER2-zero expression (17). Furthermore, alterations in HER2 status can occur over time and during treatment, transitioning from primary to recurrent BC, influenced by processes such as epithelial-mesenchymal transition and gene mutations (18).

Despite the sensitivity of DCE-MRI in detecting BC, distinguishing between HER2-low and HER2-zero expression remains challenging. Radiomics, which extracts and analyzes quantitative data from medical images, offers insights into the tumor microenvironment (19). DCE-MRI features can predict molecular subtype, histology, recurrence risk, treatment response, and HER2 status (20–24).

However, no studies have explored using radiomics to differentiate HER2-low and HER2-zero BC on DCE-MRI data. Hence, this study aims to identify specific radiomics features that can distinguish between HER2-low and HER2-zero BC, addressing a significant gap in current knowledge.

## Materials and methods

### Patient set

**Figure 1:**
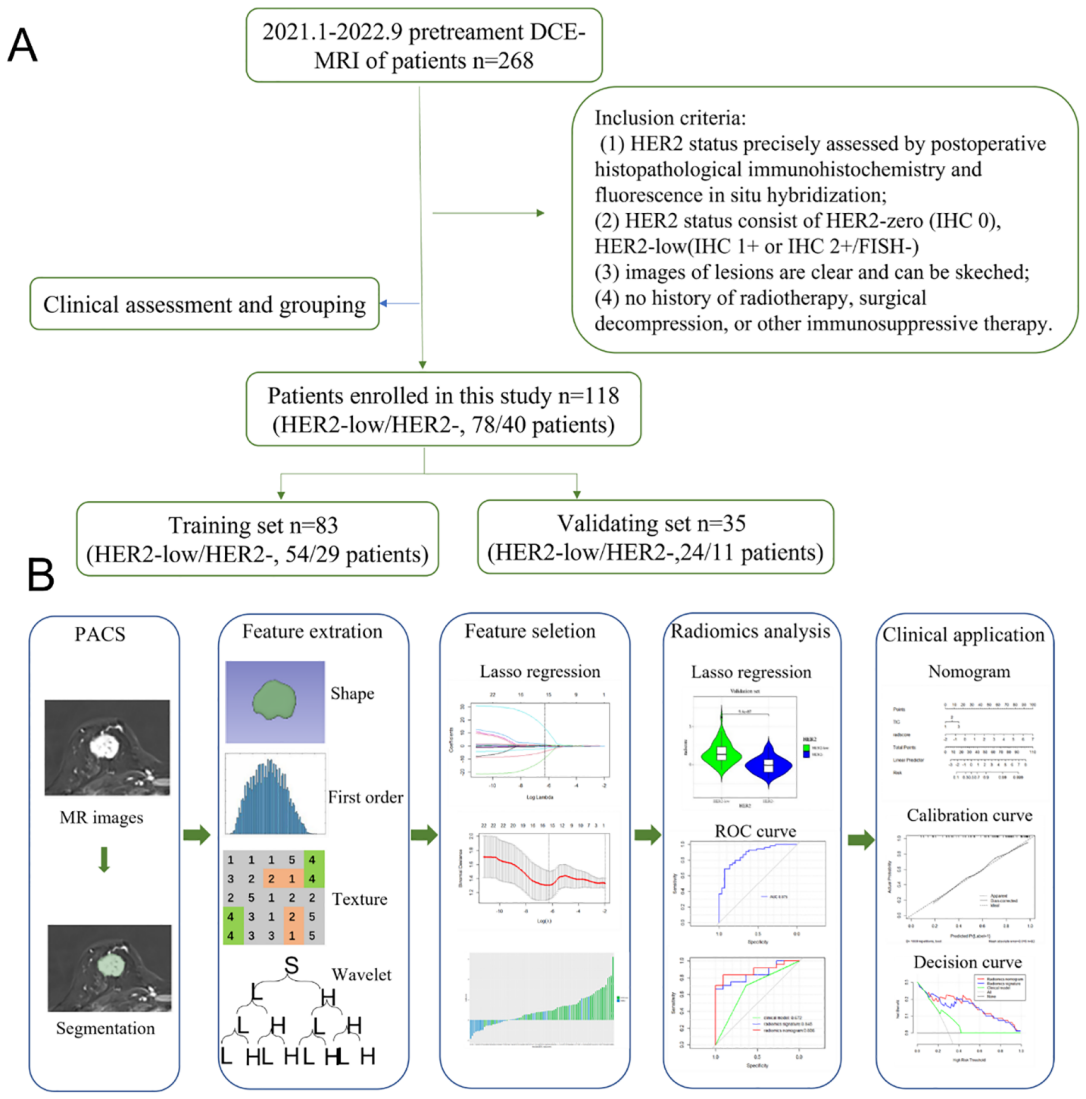
**(A)** Flowchart of the methods based on radiomics analysis for differentiation between HER2-low and HER2-zero. **(B)** The workflow of the radiomics analysis.

The Institutional Ethics Council of Jiangsu University Affiliated People’s Hospital gave its approval to this retrospective study (K-20230002-W). The methods employed in differentiating between HER2-low and HER2-zero are depicted in a flowchart in **Figure 1**. From January 2021 to September 2022, the research included a total of 568 participants who performed DCE-MRI examinations. Informed consent was obtained from all individual participants included in the study. The inclusion criteria were: (1) HER2 status precisely assessed by postoperative histopathological IHC and fluorescence *in situ* hybridization; (2) HER2 status consist of HER2-zero (IHC 0), HER2-low (IHC 1+ or IHC 2+/FISH-negative), (3) images of lesions are clear and can be sketched; (4) no history of radiotherapy, surgical decompression, or other immunosuppressive therapy. The final group included 118 BC patients (78 HER2-low breast cancer patients and 40 HER2-zero breast cancer patients) for analysis.

### HER2 status

IHC 0 was used to define HER2-zero. IHC 1+/2+ with a HER2 amplification negative result by *in situ* hybridization (ISH) methods was designated as HER2-low. The American Society of Clinical Oncology (ASCO)/College of American Pathologists (CAP) recommendations state that HER2 amplification is detected when the HER2/chromosome 17 centromere ratio is ≥2.0 (after 2013), the HER2 Copy Number is ≥6.0, or the HER2 IHC is 3+.

### MRI acquisition

A 3.0-T MRI scanner (Magnetom Skyra; Siemens Healthcare) with a specialized 16-channel breast coil was used for the breast MRIs. Fat-suppressed diffusion-weighted axial echo planar images were taken after anatomical localization (field of view = 330 mm, repetition/echo time= 6200/45 ms, matrix = 384 384 mm, slice thickness = 4 mm, 28 slices, parallel imaging factor = 2, total imaging time= 2.37 min). Diffusion weighting was used with b = 50, 800, and 1000 s/mm2. The signal was averaged after three acquisitions to improve the signal-to-noise ratio.

Following that, fat-suppressed volume-interpolated breath-hold DCE MRI images were obtained in three dimensions (3D). Six post-contrast scans were obtained after a 2.5 mL/s intravenous injection of 0.2 mL/kg Gadoterate Meglumine (Gd-DOTA), which was followed by a 13-mL saline flush with a power injector (Irich; Nemoto Kyorindo). DCE-MRI parameters were as follows: field of view = 340 mm, repetition/echo time = 4.12/1.61 ms, flip angle = 10°, matrix = 384 mm,88 sections, parallel imaging factor = 1, and total imaging duration = 44 s.

### Tumor masking and radiomic feature extraction

**Figure 2:**
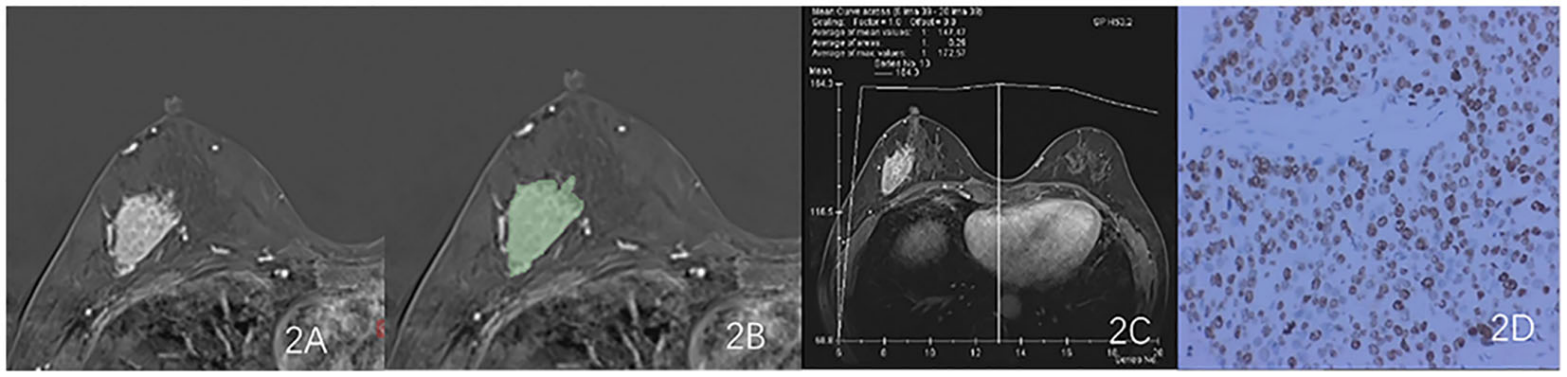
Results obtained from a randomly-selected HER2-low (HER2 2+ negative) case. **(A)** Subtraction image of pre- and post-contrast scans. **(B)** Enlarged image showing the ROI (red region) delineated manually by an experienced radiologist, then, the segmentation was examined by another radiologist. **(C)** TIC curve (platform type) of the DCE image. **(D)** Pathology results showing IDCS (HER2 2+ gene confirmed by IHC).

**Figure 3:**
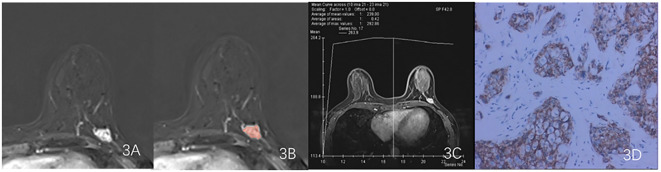
Results obtained from a randomly-selected HER2 - case. **(A)** Subtraction image of pre- and post-contrast scans. **(B)** Enlarged image showing the ROI (red region) delineated manually by an experienced radiologist, then, the segmentation was examined by another radiologist. **(C)** TIC curve (platform type) of the DCE image. **(D)** Pathology results showing IDCS (HER2- gene confirmed by IHC).

Export the DCE phase 2 images from the Picture Archiving and Communication System (PACS) in DICOM format (**Figure 1**). A radiologist, blinded to the pathological results, manually delineated the tumor regions using 3D Slicer 4.11 software, avoiding the necrotic and cystic areas of the lesion. If the patient had multiple lesions, the largest lesion was selected for delineation (**Figures 2**, **3**). Using a simple random sampling method, 16 cases were selected from 118 patients. Another radiologist, blinded to the pathological results, re-delineated the regions of interest for reproducibility analysis using the intraclass correlation coefficient (ICC).

### Feature extraction, selection method and radiomic model construction

960 features that were extracted from every ROI using Pyradiomics library (https://pyradiomics.readthedocs.io/en/latest/), covering the following categories: 14 shape features derived from the original image, 18 first-order features, 68 texture features, 36 first-order features following LOG transformation, 136 texture features, and 688 small porter features following wavelet transformation. Z-score normalization was used to standardize all of the radiomic characteristics.

**Figure 4:**
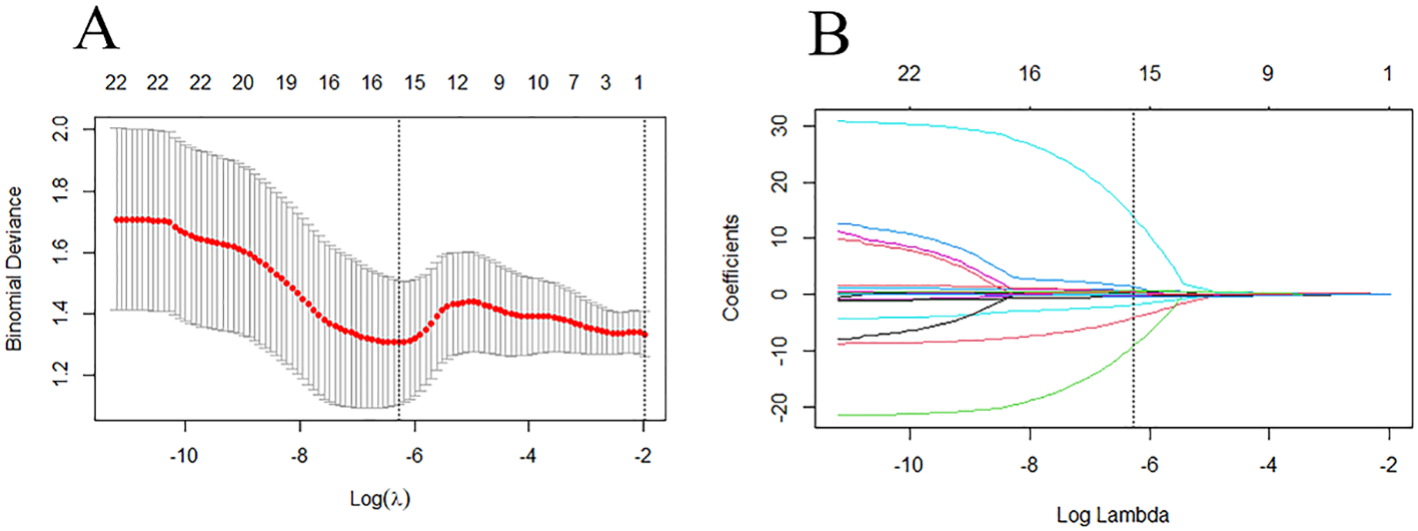
LASSO regression model was used to analyze and select the characteristics of the differentiation between HER2-low and HER2-zero. **(A)** The λ-variation chart of the adjustment parameter was selected by cross-testing of the minimum standard 10 times. The vertical line describes the chosen optimal λ value of 0.002, with log (λ) of -6.214 **(B)** Change chart of adjustment parameter λ and LASSO screening feature and 16 radiomics features with non-zero coefficients were selected.

**Figure 5:**
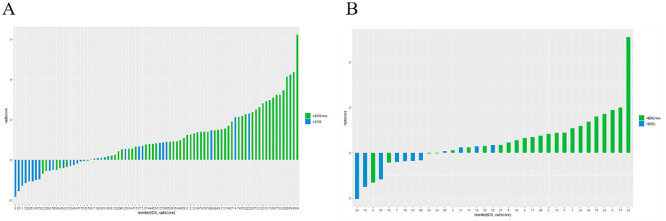
Rad-score differed significantly between the HER2-low and HER2-zero groups in both the training **(A)** and testing sets **(B)**.

The ICC was used to evaluate the consistency of radiomic features extracted from the regions of interest delineated by two radiologists in 16 patients. Only features with an ICC value greater than 0.75 were retained for further study. The features filtered through ICC analysis were subjected to the Mann-Whitney U test. Dimensionality reduction of the features was performed using the least absolute shrinkage and selection operator (LASSO) regression method with 10-fold cross-validation, identifying features that distinguish between HER2-low expression and HER2-0 (**Figure 4**). The radscore for each patient was calculated by multiplying the selected feature values by their respective weight coefficients (**Figure 5**). Using logistic regression analysis method, the radiomics model was constructed based on the best eigenvalues of DCE-2 images.

### Clinicopathological MRI feature modelling

To construct the clinicopathological MRI feature model, patient data including age, maximum diameter of the tumor, clinical stage, lymph node metastasis status, type of time intensity curve (TIC), ER expression, PR expression, and Ki-67 index were collected. Continuous clinical feature variables were analyzed using t-tests, and categorical clinical feature variables were analyzed using chi-square tests or Fisher’s exact tests. Multivariate binary logistic regression analysis was performed on the statistically significant clinico-pathological mri features in the training set to determine the correlation between each feature and HER-2 expression status in breast cancer. Statistically significant variables were then selected to construct the clinico-pathological MRI feature model.

### Radiomic nomogram construction and evaluation

Using the optimal radiomics features from the training set combined with statistically significant clinicopathological MRI features, a nomogram model was constructed and validated with the validation set. The calibration curves were plotted to evaluate the nomogram’s diagnostic accuracy in both sets. The Hosmer-Lemeshow goodness-of-fit test was used to calibrate the curve and evaluate the success of the nomogram. “The clinical application of the model in predicting the pretreatment therapeutic response based on the differentiation between HER2-low and HER2-zero breast cancer was evaluated using decision curve analysis.

### Statistical methods

R3.3.1 and SPSS 26.0 software were used for statistical analysis. Measurements conforming to the normal distribution are expressed as $\bar{\text{x}}$ ± s. Comparison between groups of count data was conducted using chi-square test or Fisher’s exact test. Multivariate logistic regression was then used to evaluate the relationship between clinico-pathological MRI features of breast cancer and HER-2 status in the training set. The classification performance of radiomics features was measured using the area under the receiver operating characteristic curve (AUC) and the 95% confidence interval (95% CI). Sensitivity, specificity, and accuracy were calculated at the point where the Youden index was maximized.

## Results

### Clinicopathological characteristics

Among the 118 enrolled patients, 78 patients were identified as HER2-low, and 40 patients were HER2-zero. The allocated training set included 54 HER2-low and 29 HER2-zero patients, and the testing set included 24 HER2-low and 11 HER2-zero patients.

Clinicopathological characteristics included age, maximum diameter, ALN status, clinical stage, DCE-MRI TIC, and IHC findings. In the training set, TIC curve (P=0.002), ER status(P=0.031), PR status(P=0.025) showed statistically significant differences between HER2-low and HER2-zero patients. For the other characteristics, there were no significant difference between the HER2-low and HER2-zero categories in the two datasets (**Table 1**).

**Table 1:** Comparison of clinical features between the training set and the testing set.

Characteristic	Training Set		p	Testing Set		p
	HER2-low	HER2-		HER2-low	HER2-	
**Age(year)**	54.4 ± 12.0	51.9 ± 12.5	0.384	55.0 ± 10.4	54.4 ± 9.9	0.874
**Maximum Diameter(mm)**	22.1 ± 10.6	23.7 ± 13.2	0.543	21.7 ± 8.4	18.2 ± 10.3	0.293
Clinical Stage						
I	4	1	0.539	2	1	0.541
II	19	8		12	6	
III	31	20		10	4	
ALN status						
Nonmetastatic	42	20	0.238	19	7	0.371
Metastatic	10	5		5	4	
ER						
Positive	45	18	0.031	18	8	0.886
Negative	9	11		6	3	
PR						
Positive	41	15	0.025	16	8	0.720
Negative	13	14		8	3	
Ki-67 status						
High(≥14%)	38	20	0.894	15	9	0.252
Low(<14%)	16	9		9	2	
DCE-MRI TIC						
I	8	5	0.002	2	2	0.155
II	16	19		5	5	
III	30	5		17	4	

DCE-MRI, Dynamic contrast-enhanced resonance; TIC, Time intensity curve; ER, Estrogen receptor; PR, Progestone receptor.

### Clinical model

According to tab2, TIC (p=0.002), ER status (P=0.031), and PR status (P=0.025) were all significantly associated with HER2-low status in the training set. The multivariate analysis revealed that TIC was an independent clinic-radiological risk factor for HER2 (**Table 2**). To construct a clinicopathological MRI feature model, ROC curve analysis showed that the AUC for predicting HER-2 expression status in breast cancer using the clinicopathological MRI feature model was 0.692 (95% CI:0.584, 0.798) in the training set and 0.675 (95% CI:0.491, 0.854) in the test set, respectively (**Table 3**).

**Table 2:** Multivariate logistic regression analysis of clinicopathological MRI features in training set.

factor	OR (95%CI)	*P*
DCE-MRI TIC		
**I**	1	0.026
**II**	0.859(0.190,3.889)	0.843
**III**	6.271(1.078,36.468)	0.041
**ER**	1.683(0.249,11.374)	0.593
**PR**	2.556(0.476,13.721)	0.274
**Radiomics score**	3.385(1.686,6.798)	0.001

ER, estrogen receptor; PR, progestone receptor; DCE-MRI, Dynamic contrast-enhanced resonance; TIC, Time intensity curve.

**Table 3:** Comparison of predictive performance between training set and testing set model.

Model	Training set				Testing set			
	AUC* (95%)	Accurary	Sensitivity	Specificity	AUC* (95%)	Accurary	Sensitivity	Specificity
T1WI model	0.875(0.800-0.951)	0.771	0.685	0.931	0.845(0.717-0.972)	0.686	0.542	1.000
Clinic model	0.691(0.584-0.798)	0.675	0.574	0.862	0.672(0.491-0.854)	0.686	0.708	0.636
Combined model	0.892(0.853-0.962)	0.807	0.796	0.828	0.886(0.777-0.996)	0.857	0.833	0.909

^*^ AUC, area under the ROC, curve.

### Radiomics features of HER2-low and HER2-zero

Logistic regression was used to model the final sixteen radiomics features (**Table 4**). The ROC curve was used to evaluate the model’s efficacy.

**Table 4:** Sixteen radiomic features were finally screened by LASSO regression.

Number	Features	coefficient
1	original_gldm_DependenceVariance	0.15336797
2	wavelet.LHL_glcm_InverseVariance	0.04272137
3	wavelet.LHH_firstorder_Skewness	0.45739730
4	wavelet.HLH_glrlm_LowGrayLevelRunEmphasis	1.36739971
5	wavelet.HLH_glrlm_ShortRunLowGrayLevelEmphasis	-1.79856221
6	wavelet.HHL_firstorder_90Percentile	-4.18110675
7	wavelet.HHL_firstorder_InterquartileRange	-9.21333167
8	wavelet.HHL_firstorder_Kurtosis	0.78092404
9	wavelet.HHL_firstorder_RobustMeanAbsoluteDeviation	13.86883477
10	wavelet.HHH_glcm_ClusterProminence	0.42960439
11	wavelet.HHH_glrlm_ShortRunLowGrayLevelEmphasis	0.65581663
12	wavelet.HHH_gldm_LargeDependenceHighGrayLevelEmphasis	-0.36074322
13	wavelet.HHH_glcm_MaximumProbability	-0.10348222
14	wavelet.HHH_glrlm_LongRunHighGrayLevelEmphasis	-0.37411624
15	wavelet.HLH_gldm_LowGrayLevelEmphasis	0.08949765
16	wavelet.HHH_glrlm_ShortRunEmphasis	0.36279122

GLDM, A Gray Level Dependence Matrix; GLCM, Gray Level Co-occurrence Matrix; GLRLM, A Gray Level Run Length Matrix.

**Figure 6:**
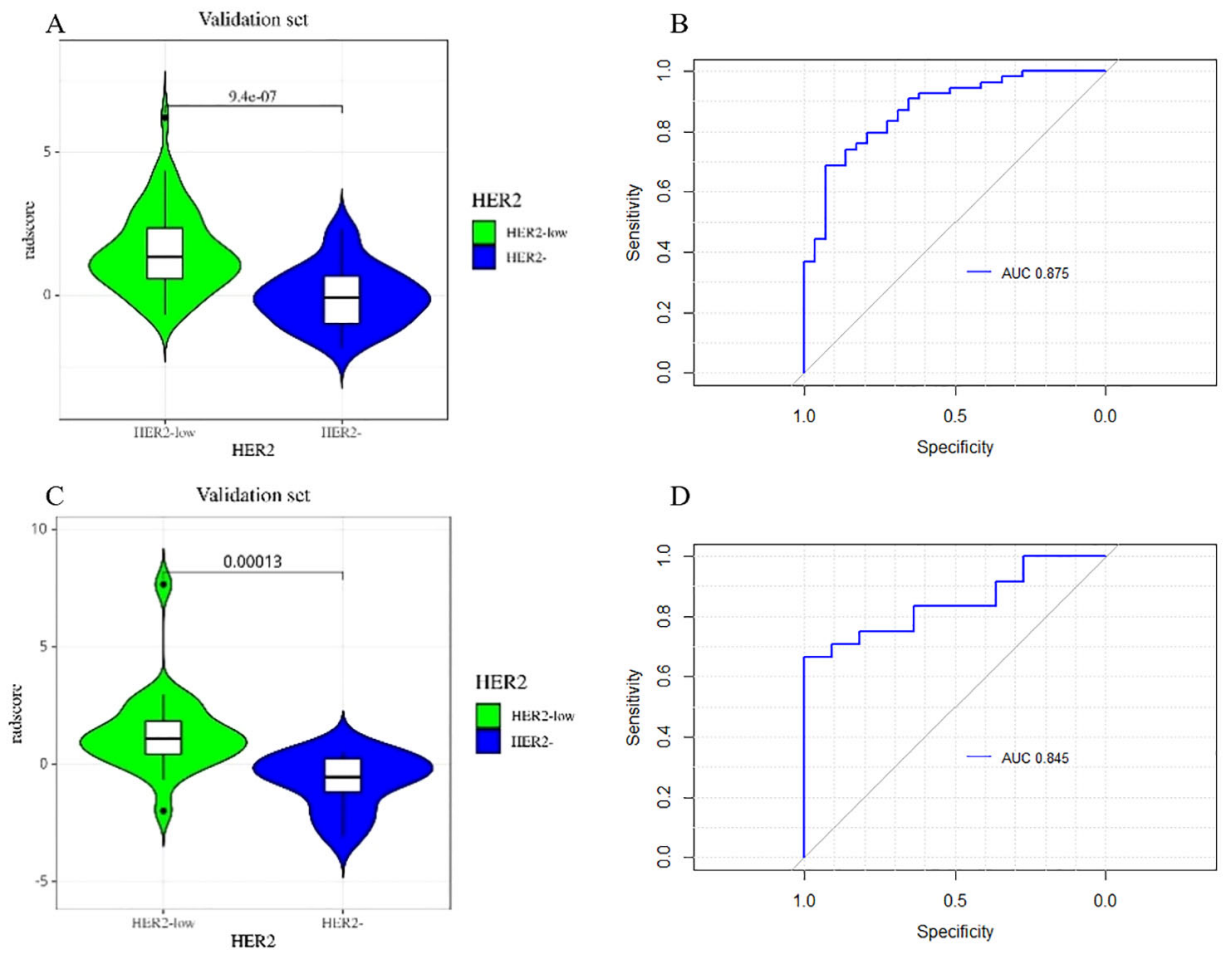
The violin plot of the radscore in the final model for the prediction of HER2-low status. Violin plots of the radiomics score (Rad-score) in the training set **(A)** and testing set **(C)** (p < 0.05). ROC curves of training set **(B)** and testing set **(D)** based on the radiomics model.

**Figure 6** depicts the ROC and violin plots for the training and testing sets. When the Jorden index is maximum, the best cutoff value is 0.673. The radiomics model’s AUC, sensitivity, and specificity for preoperative HER2-low were 0.875 (95% CI:0.800,0.951), 68.5%, and 93.1% in the training set and 0.845 (95% CI:0.717,0.972), 54.2%, and 100.0% in the testing set, respectively. Rad-scores differed significantly in both the training (a) and testing sets between the HER2-low and HER2- groups (b).

### Development and testing of radiomics nomogram

**Figure 7:**
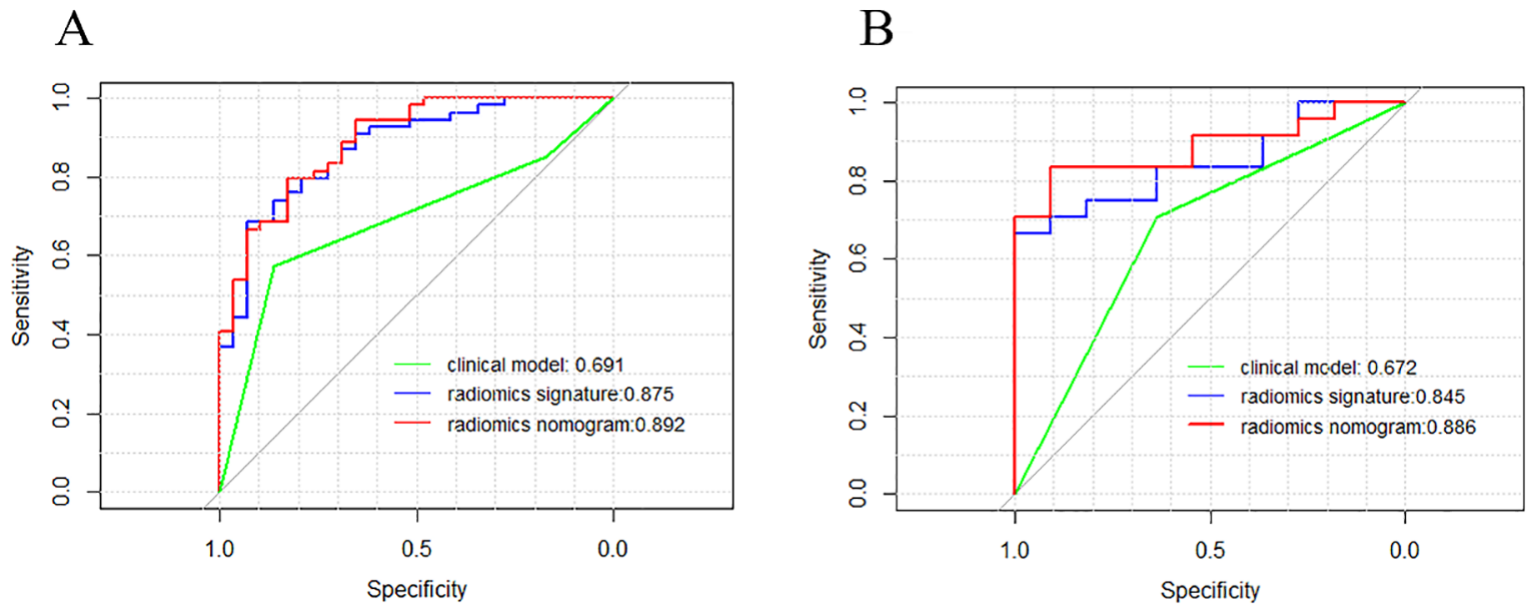
Comparison of receiver operating characteristic (ROC) curves of the clinical model (green line), the radiomics signature (blue line), and the combined clinical nomogram (red line) for the prediction of difference between HER2-low and HER2- in the training **(A)** and testing **(B)** sets, respectively.

**Figure 8:**
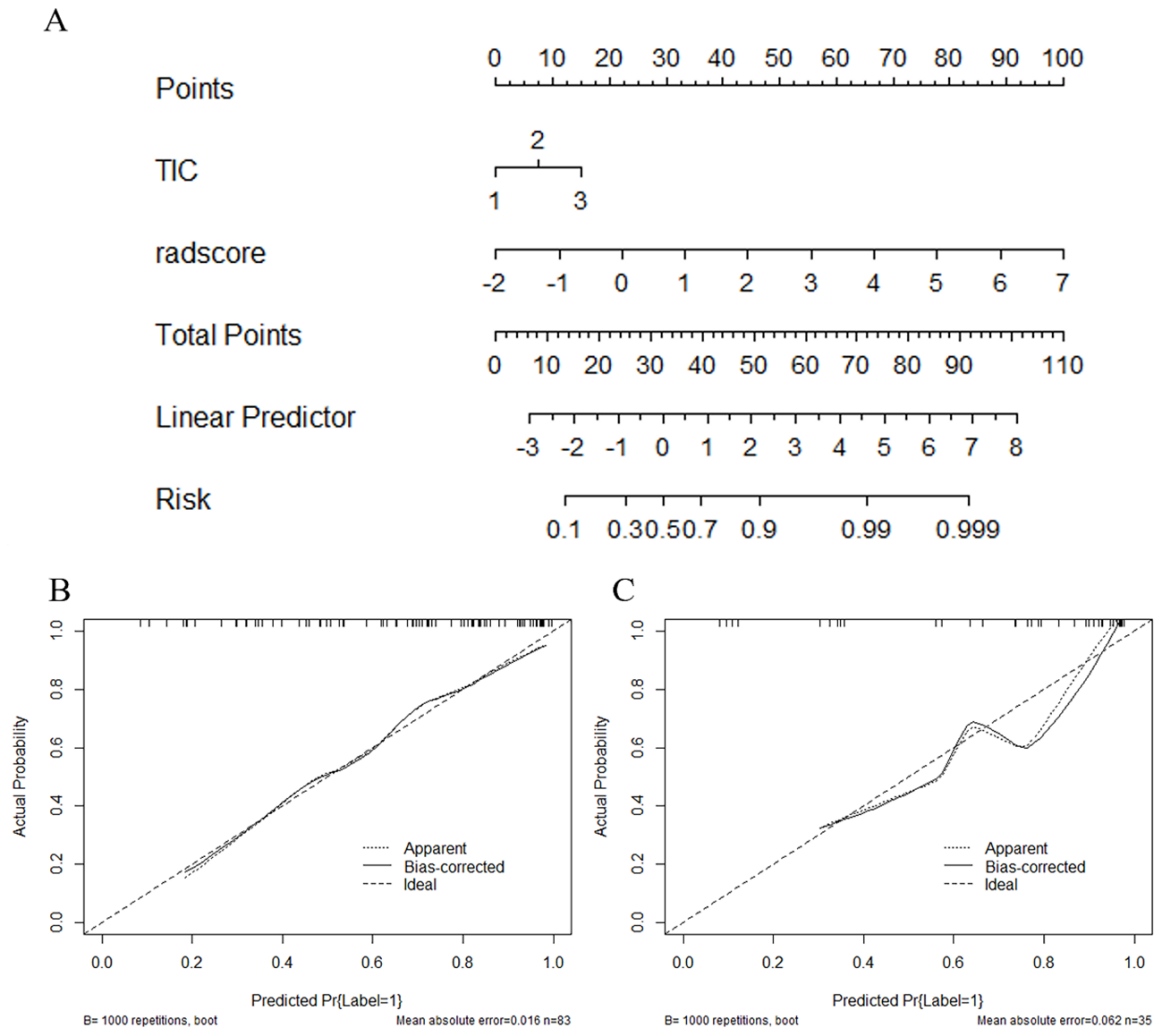
The combined radiomics nomogram for the prediction of HER2 in breast cancer patients and the calibration curve of the nomogram. **(A)** The combined radiomics nomogram established by incorporating the TIC curve and the radiomics score **(B, C)**. The calibration curves of the nomogram in training and testing sets. The X-axis represents a nomogram predicted probability of HER2, the Y-axis an actual HER2-low status, and the diagonal dashed line indicates the ideal prediction by a perfect model.

**Figure 7** depicts a combined radiomics nomogram created by incorporating significant clinical radiological risk factors and radscore into the HER2 prediction algorithm. The nomogram demonstrated superior predictive performance with AUCs of 0.892 (95% CI: 0.853-0.962) and 0.886 (95% CI: 0.777-0.996) for both sets, respectively. Also, it outperformed the models incorporating only the clinic-radiological semantic features or the radiomics signature (**Table 4**). **Figure 8** represents the combined radiomics nomogram for predicting HER2 in BC patients, as well as the nomogram’s calibration curve.

**Figure 9:**
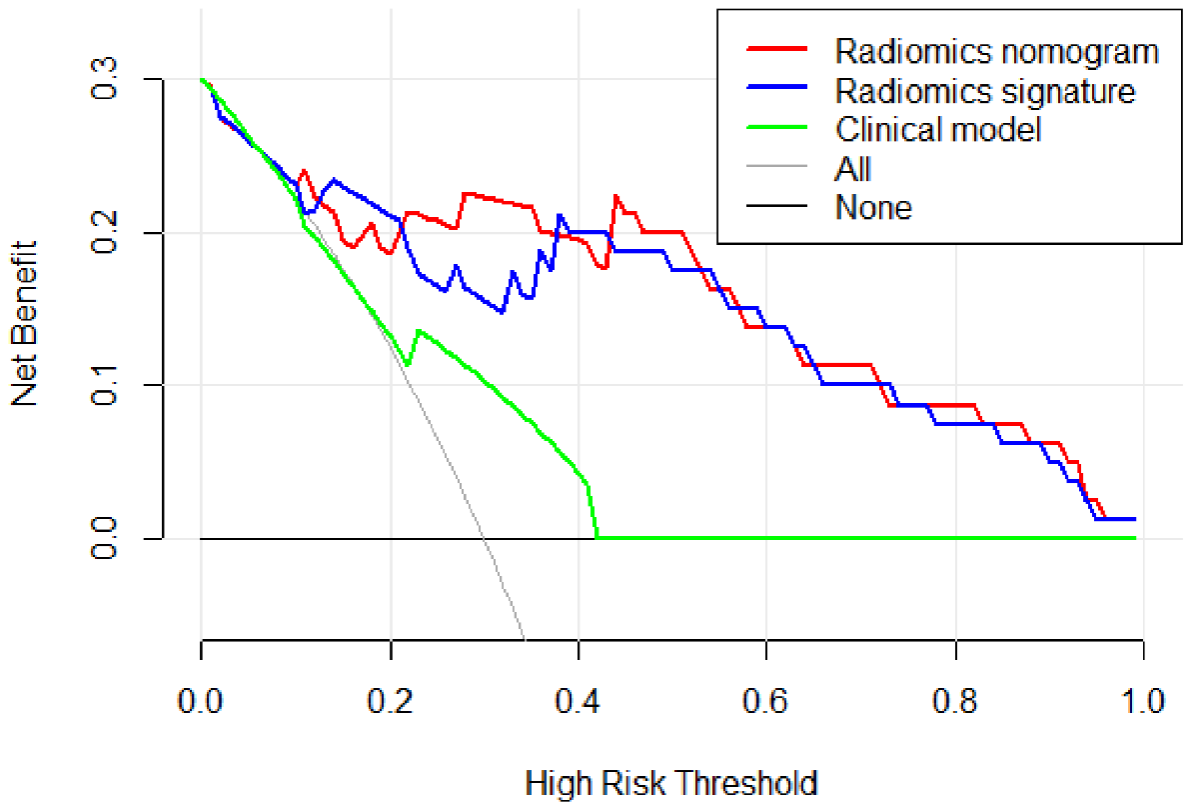
The decision curve analysis (DCA) of the clinical model (green line), the radiomics signature (blue line), and the combined radiomics nomogram (red line) in the testing set. It depicts the clinical model’s DCA, the merged radiomics nomogram, and the radiomics signature. The combined radiomics nomogram outperformed either the clinical-radiological features or the radiomics signature alone in the 20%-38% potential range.

**Figure 9** describes the clinical model’s Decision Curve Analysis (DCA), the merged radiomics nomogram, and the radiomics signature. The combined radiomics nomogram outperformed both the clinical-radiological features and the radiomics signature alone in the 20%-38% potential range.

The Delong test compares the prediction of the combined model with the clinical model’s measured effectiveness (AUC value), and the results revealed a statistical difference in the training set (P <0. 01) and testing set (P = 0. 01). The Delong test compares logistic regression model prediction to clinical model measured effectiveness (AUC value). The results showed a statistical difference in the training set (P <0. 05), but no statistical difference in the test set (P = 0.105).

## Discussion

In our comprehensive study, we conducted an extensive examination of clinical and radiomics characteristics with the aim of predicting HER2-low and HER2-zero status in BC patients. Our findings revealed that when incorporated into predictive models, TIC, ER, and PR status exhibited significant contributions, complemented by radiomics features. The integration of these factors into a unified radiomics nomogram has yielded a substantially enhanced predictive tool, holding considerable promise for its potential clinical applicability in tailoring personalized treatment strategies for HER2-low BC patients.

In approximately 60% to 70% of breast tumors, the HER2 receptor protein is detectably expressed by IHC, with a HER2 IHC score of 1+ and above (25, 26). Studies reveal that score 3+ cells have about two million HER2 receptor molecules on their membrane, while score 1+ and 2+ BC cells have 100,000 to 500,000 HER2 receptor molecules (27). Recently, a subset of breast cancers with low HER2 expression and no apparent ERBB2 amplification has been approved for novel anti-HER2 medicines, particularly HER2-targeting ADCs (28). This subset, referred to as “HER2-low” BC, is characterized by HER2 with IHC 1+ or 2+ and negative ISH (29). For instance, trastuzumab deruxtecan (DS8201a) demonstrated an ORR of 37% in extensively pretreated patients with HER2-low metastatic BC (13). Given their distinct biology, treatment responses, and clinical outcomes, HER2-low tumors should be recognized as a novel BC subtype separate from HER2-zero (IHC 0) tumors. In the future, the definition of HER2 status in BC will comprise a three-step approach including i) HER2 positive, ii) HER2-negative, and iii) HER2-low breast carcinomas, with the latter potentially benefiting from targeted treatment options.

Several studies have demonstrated the association between classic imaging modalities like mammography (MG) and ultrasound (US) with the HER2 status in BC patients. For instance, breast density and a spiculated mass on MG have been linked to HER2 status (29–31). However, the predictive power of these variables for determining HER2 status is limited. In contrast, MRI methods offer a more effective means to identify heterogeneity in a quick, direct, and non-invasive manner (32). Dynamic contrast-enhanced images, due to the increased permeability in tumor tissue, provide valuable information on the tumor’s morphology (shape, size, and extension), kinetic contrast agent uptake, angiogenesis, and prognostic features such as type and grade (33).

AI-based models have demonstrated remarkable accuracy in cancer prediction and therapeutic efficacy based on genetics and hormonal parameters (34, 35). Additionally, recent research by Chen and Guo et al. utilized PET/CT, X-ray, MR, and radiomics to predict HER2 expression (36, 37). Notably, MRI-based machine learning radiomics has shown promise in predicting HER2 expression levels and pathologic response following neoadjuvant treatment in HER2 overexpressing BC (38). Furthermore, Jiandong Yin et al. reported that semiquantitative kinetic parameter maps of HER2-positive BC exhibit greater heterogeneity and texture complexity compared to HER2-negative BC, serving as potential imaging biomarkers to distinguish between the two subtypes (23). Despite this progress, the distinction between HER2-low and HER2-zero BC had not been addressed in radiological research until now. In this study, HER2 status was determined using IHC and FISH, and radiological features from DCE-MRI were employed to differentiate between HER2-low and HER2-zero states. The logistic regression (LR) models achieved favorable AUC values in the training set (0.875, 95% CI: 0.833-0.966) and testing set (0.845, 95% CI: 0.661-0.979), indicating that radiological analysis using DCE-MRI images can effectively distinguish between HER2-low and HER2-zero BC subtypes. These findings highlight the potential of DCE-MRI radiomics as a valuable tool for HER2 status assessment in BC patients.

In this study, a radiomics model based on radiation characteristics was utilized to screen for 16 radiology parameters related to HER2 expression. Notably, the coefficients of gray level run length matrix (GLRLM) Low Gray Level Run Emphasis, GLRLM Short Run Low Gray Level Emphasis, first order 90 Percentile, first order Interquartile Range, and first order Robust Mean Absolute Deviation (rMAD) were found to be higher. The GLRLM was used to quantify the consecutive pixel runs with the same gray value, representing the length of the pixel number. The positive correlation between HER2-low BC and the pixel number’s length suggests differences in tumor aggressiveness and cell growth rates (39).

Furthermore, the skewness of first-order statistics based on histology of histograms was employed to quantify tumor heterogeneity, considering the average asymmetrical grayscale distribution. Higher frequency asymmetry in the grayscale distribution indicates greater tumor heterogeneity and contributes to distinguishing between HER2-low and HER2-negative tumors, potentially influenced by factors such as cell proliferation time, necrosis, and microcalcification (40).

These findings underscore the significance of the radiomics approach in discerning and comprehending the distinctions between HER2-low and HER2-negative BC, shedding light on potential factors contributing to tumor heterogeneity and aggressiveness.

In this study, dynamic enhanced radiomic features were utilized to assess their predictive efficacy for determining the HER2-low expression state. Alongside this analysis, important clinical information such as age and conventional MRI imaging features like size and TIC type, were included. Pathological information, including ER, PR, and ki67 expression, was also considered for joint analysis with dynamic enhanced radiomic features. The results indicated that the TIC type in conventional MRI imaging characteristics was associated with HER2 expression at low and zero levels. The clinical model achieved AUC values of 0.675 and 0.692 on the training and test sets, respectively. Notably, the TIC platform type and outflow type were found to be closely related to vascular permeability and angiogenesis in BC. Previous studies have highlighted that HER2-positive BC can stimulate tumor neovascularization and enhance vascular permeability through the induction of endodermal growth factors (41). Moreover, the early enhancement rate in tumors can reflect their blood supply, making the TIC platform type and outflow type more common in HER2-low BC (42).

In comparison to a single radiomic feature, the development of a nomogram that combines the LR radiomic feature and TIC curve demonstrated an improvement in distinguishing between HER2-low and HER2-zero states. The predictive AUC of the model increased from 0.875 to 0.892 in the training set and from 0.845 to 0.886 in the test set. This suggests that incorporating conventional imaging features with radiomics features can further enhance the model’s effectiveness. This approach simplifies complexity and enhances the practicality and extensibility of the model, providing potential benefits in clinical application.

The current study has several limitations. Firstly, it is retrospective, small-scale, and conducted at a single center, which may limit the generalizability of the results and the ability to accurately predict the difference between HER2-low and HER2-zero BC. To improve discrimination between HER2-zero and HER2-low, future studies should consider utilizing additional neighboring slices at the 3D level, as the current analysis was limited to one slice image (2D). Moreover, while the prediction model used the commonly used LR model, it was not compared with other widely-used machine learning algorithms such as SVM, random forest, and decision tree. Further investigations are needed to explore whether other methods can achieve models with better prediction efficiency.

## Conclusion

This study highlights the promising potential of using radiomic features from breast DCE-MRI to differentiate between HER2-low and HER2-zero BC. The findings suggest that this approach could serve as a non-invasive and practical tool for predicting HER2-low BC preoperatively, assisting oncologists in making clinical decisions. However, to validate and further enhance the reliability of these findings, larger sample sizes and prospective randomized trials are necessary in future research endeavors.

## Data Availability

The raw data supporting the conclusions of this article will be made available by the authors, without undue reservation.
